# Promising Antibiofilm Activity of Peptidomimetics

**DOI:** 10.3389/fmicb.2018.02157

**Published:** 2018-09-13

**Authors:** Rafael Gomes Von Borowski, Simone Cristina Baggio Gnoatto, Alexandre José Macedo, Reynald Gillet

**Affiliations:** ^1^Univ Rennes, CNRS, Institut de Génétique et Développement de Rennes (IGDR), UMR 6290, Rennes, France; ^2^Programa de Pós-Graduação em Ciências Farmacêuticas, Faculdade de Farmácia, Biotechnology Center, Universidade Federal do Rio Grande do Sul, Porto Alegre, Brazil

**Keywords:** antibiotic resistance, biofilm, peptides, peptidomimetics, AApeptides, peptoids

## Abstract

Pathogenic biofilms are a global health care concern, as they can cause extensive antibiotic resistance, morbidity, mortality, and thereby substantial economic loss. Scientific efforts have been made over the past few decades, but so far there is no effective treatment targeting the bacteria in biofilms. Antimicrobial peptidomimetics have been proposed as promising potential anti-biofilm agents. Indeed, these structurally enhanced molecules can mimic the action of peptides but are not susceptible to proteolysis or immunogenicity, the characteristic limitations of natural peptides. Here, we provide insights into antibiofilm peptidomimetic strategies and molecular targets, and discuss the design of two major peptidomimetics classes: AApeptides (*N*-acylated-*N*-aminoethyl-substituted peptides) and peptoids (*N*-substituted glycine units). In particular, we present details of their structural diversity and discuss the possible improvements that can be implemented in order to develop antibiofilm drug alternatives.

## Introduction

The increased resistance of biofilms to antibiotics is a global health care problem (Costerton et al., 1999; Hall and Mah, 2017). Biofilms are well-organized microbial clusters which produce a matrix from a series of compounds that include extracellular DNA (eDNA), proteins, and polysaccharides. These compounds are either attached to a surface (when originating on medical devices or teeth) or are suspended (in mucus or in chronic wounds) (Flemming and Wingender, 2010). Their form confers advantages over planktonic cells to the matrix-enclosed microorganisms, including improved biocide tolerance, host immune defense, and persistence. These advantages are caused by vast physiological and biochemical changes, including slow cell growth, beneficial quorum sensing, and higher mutation rates (Davies, 2003). Indeed, chronic bacterial infections are themselves encouraged by the accumulation of bacteria in the biofilm-producing biopolymer matrix. Since they are embedded into the matrix, these bacteria have an increased tolerance to antibiotics, chemical disinfectants, and/or host defenses, and are much harder to treat than infections without biofilm (Høiby et al., 2010; Beloin et al., 2014).

The most relevant clinical biofilm-forming bacteria are the gram-negative *Acinetobacter baumannii, Escherichia coli, Klebsiella pneumoniae*, and *Pseudomonas aeruginosa*, along with gram-positive *Staphylococcus aureus* and the less virulent *S. epidermidis* (Jabbouri and Sadovskaya, 2010; de la Fuente-Núñez et al., 2013; Chen et al., 2014; Culotti and Packman, 2014; Longo et al., 2014; Andrea et al., 2018). These microorganisms can form biofilms on virtually any medical device, including cardiac pacemakers and prosthetic heart valves, endotracheal tubes, urinary catheters, central venous catheters, prostheses, orthopedic devices, contact lenses, and dentures (Baquero and Coque, 2011). This ability is possible due the broad genetic variability of the microbial populations found in health care institutions. This genetic spectrum, also implying phenotypic variations, occurs within the same species. This makes it difficult to develop a therapy or even a general surface material that could deter the growth and adhesion of these microorganisms (Cegelski et al., 2008). Medical devices are an important cause of human infections, for instance turning *S. epidermidis* into an important emerging pathogen responsible for most infections in central venous catheters. This results in the need to remove and replace the medical device, increasing costs and patient suffering (Maki et al., 2006). Not only do bacteria have the individual capacity to form biofilm, but in biofilm some strains will have increase their horizontal transfers of plasmids carrying antibiotic resistance genes, thus increasing mutation frequency (Savage et al., 2013). For all of these reasons, pathogenic biofilms have a huge clinical impact in terms of economic losses, morbidity, and mortality.

Therefore, bacterial biofilms are promising targets for combatting this problem of antibiotic resistance. The successful development of antibiofilm compounds will therefore be an important tool for controlling human infections (Miquel et al., 2016).

In this context, peptides have been proposed as an important direction to follow, either for creating alternative drug therapies or for developing new anti-infective surfaces (Riool et al., 2017).

Peptides are fundamental molecules made up of 2–50 amino acids, with many biological functions. Indeed, their versatile chemical features such as malleability and multifunctionality, make them good models for the synthesis of new bioactive compounds (Von Borowski et al., 2017). Antimicrobial peptides (AMPs) are very interesting molecules to be explored in the search for antibiofilm agents to replace conventional antibiotics. This is because they are relatively easy to produce while exhibiting broad-spectrum antimicrobial activity, with a distinct mode of action that means that they are less prone to developing resistance (de la Fuente-Núñez et al., 2013; Strempel et al., 2015; Andrea et al., 2018). However, although natural peptides are indispensable for the structure, functioning, and metabolism of each living organism, their regulation is mediated by molecular interactions, proteolysis, and immunogenic responses (Avan et al., 2014). Thus low stability and availability limits their therapeutic relevance. On the other hand, peptidomimetics are chemically modified expressly to limit the drawbacks of natural peptides. The underlying strategy is to create small peptide-like molecules that still have the inherent abilities of natural ones (so that the advantageous biological effects remain), but which are more stable and available, with improved selectivity and/or potency (Grauer and König, 2009; Croft and Purcell, 2011). In this context, rather than joining the amino acids to a bioisosteric group to mimic the original amide, a very efficient chemical strategy is to replace the peptide bond, the -CO-NH- amide (Niu et al., 2013). Although quite a number of amide bond replacements have been reported, our review focuses here on the *N*-acylated-*N*-aminoethyl amino acids (AApeptides) and on peptoids. These specific strategies were chosen as they each have pronounced chemical diversity, can mimic both the primary and secondary structures of peptides, resist proteolysis, and show good activity against pathogenic biofilms. By presenting these promising candidates, we pave the way for the design of more active and safer innovative molecules.

## Peptidomimetics are an improvement over natural peptides

Despite their inherent robust and promising bioactivity, there are drawbacks to the use of natural peptides, including their high clearance and their susceptibility to proteolysis or immunogenicity, both of which can cause unwanted effects (Von Borowski et al., 2017). Inspired by natural peptides, chemists have developed a variety of structurally diverse synthetic mimics with key physicochemical natures (*i.e*., cationic charges and amphiphilicity) which they call *peptidomimetics*.

These molecules can be obtained in different ways. Peptidomimetics can be made by manipulating the amino acid backbone of native peptides in order to enrich structural diversity, making them extraordinarily useful. They can also be prepared through the coupling of stable unnatural amino acids generated via modifications such as amine alkylation. For example, poly-N-substituted glycines allow for the generation of peptoids that differ from peptides only in their side chains, making them protease-resistant (Miller et al., 1995). Another strategy is the isosteric replacement of the amino group by for example an oxygen or sulfur atom. This changes the H-bonding pattern, significantly affecting the secondary structure and folding properties of peptides. Another possible approach for getting peptidomimetics that have new secondary structures and biological activities involves taking natural peptides and performing C-α configuration inversion, α-hydrogen replacement (by the alkyl or other groups), and replacing the α-carbon atom by heteroatom, mostly nitrogen (Avan et al., 2014). Several of these strategies have been used to synthesize peptidomimetics which appear to be as promising against biofilms as naturally occurring AMPs. In fact, many synthetic antibacterial peptidomimetics are currently undergoing clinical trials, including the membrane-disrupting compound LTX-109, the cationic steroid compound CSA-13, and the novel peptidomimetic brilacidin (see https://clinicaltrials.gov/). Recently, short peptidomimetics made of Arg and N-alkyl/aryl pyrazole residues were shown to have good antimicrobial and anti-inflammatory activities, increased proteolytic stability against trypsin digestion, and antimicrobial activity, even in the presence of physiological salts (Ahn et al., 2017). Another series of AMP mimetics were synthesized by incorporating a 3′-amino-[1,1′-biphenyl]-3-carboxylic acid backbone in MSI-78, a peptide currently in Phase-III clinical trials (Kuppusamy et al., 2018).

### AApeptides as a peptidomimetic strategy

**Figure 1:**
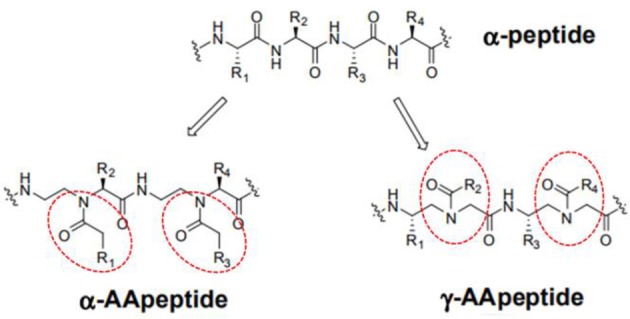
Structure illustration of an α-peptide and its corresponding AApeptide models, adapted from Niu et al. (2013). The α- and γ- *N*-acylated-*N*-aminoethyl amino acid amide bond replacement structures are identified by dotted circles in red. R corresponds to possible radicals.

*AApeptides* are oligomers of *N*-acylated-*N*-aminoethyl-substituted amino acids that are derived from chiral peptide nucleic acid (PNA) backbones (Shi et al., 2016). The chiral side chain is connected to either the α-C or γ-C of the carbonyl group, while acylation is used to introduce the other side chain to the central N, as illustrated in Figure 1 (Sang et al., 2017). Compared to their original peptide counterparts, AApeptides have the same backbone lengths and functional group counts, and the same number of nitrogen atoms involved in secondary or tertiary amide bonds. In addition, they mimic the original amino acid side-chain positions, so they have the same activity. However, their backbones are more flexible, and since the AApeptides have tertiary amide bonds that can be involved in cis/trans configurations, they should have interesting hydrogen bonding properties and conformational flexibilities (Niu et al., 2013).

#### Antibiofilm AApeptides

In a recent study, Teng et al. (2016) described an acyclic model based on host defense peptides (HDPs) with charged and non-charged radicals (Table 1, ID 1). The global structure has a cationic hydrophobic residue composed of ornithine, and adamantyl or aromatic rings. This model was used to produce a global amphiphilic AApeptide that targets membrane disruption for antibiotic activity. The molecule displays high Gram-negative antifouling activity and low cytotoxicity effects. In addition, it was hypothesized that if radical 1 (R1) was a cationic group, and R2, R3, and R4 were hydrophobic, the global structure should be both an HDP and amphipathic, which will result in the killing of bacteria via membrane disruption. Accordingly, the hydrophobicities of R2, R3, and R4 were modified by inserting various groups (such as adamantyl, biphenyl, CF3, t-butyl) into the aromatic rings, then testing the resulting compounds against clinically relevant bacteria. A reduction in R2–R4 hydrophobicity correlates to decreased molecule killing capacities.

**Table 1:** Summary of chemical and biological information on AApeptides.

	**AA peptides**				Concentration^*^(μ**g/mL)/action range (%)**		Mechanism^*^				**References**

**ID**	**Peptidomimetic**	**Chemical structure**	**Molecular weight (*****M*****)**	Model^*^	**Antifouling**	**Eradication**	**Biofilm**	**Antimicrobial**	**MIC (**μ**g/mL)**	**Cytotoxicity (**μ**g/mL)**	
1	Acyclic model and the main compound 13	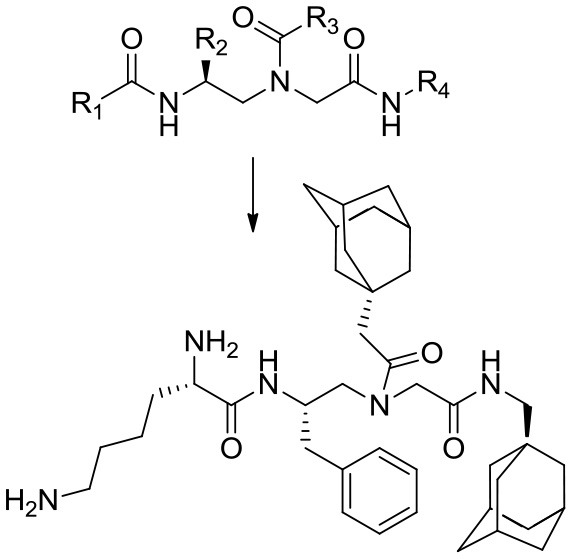 Compound 13 (C_40_H_62_N_5_O_3_) R1, cationic and R2,3,4 hydrophobic	660.4855	*Escherichia coli* (ATCC 25922), *Acinetobacter baumannii*	0.6–2/50–~75		Not shown	Membrane disruption	3.12 (*E.coli*)	(Hemolysis) 85; (HK-2) 86; (K562) 83	Teng et al., 2016
	SAR: antibacterial activity was enhanced by increasing R4's hydrophobicity. Antibacterial and hemolytic activities were decreased through the introduction of cationic charges (*K*) at R1. No SAR or other correlation to antibiofilm activity was shown.										
2	The main compound **YL-36**	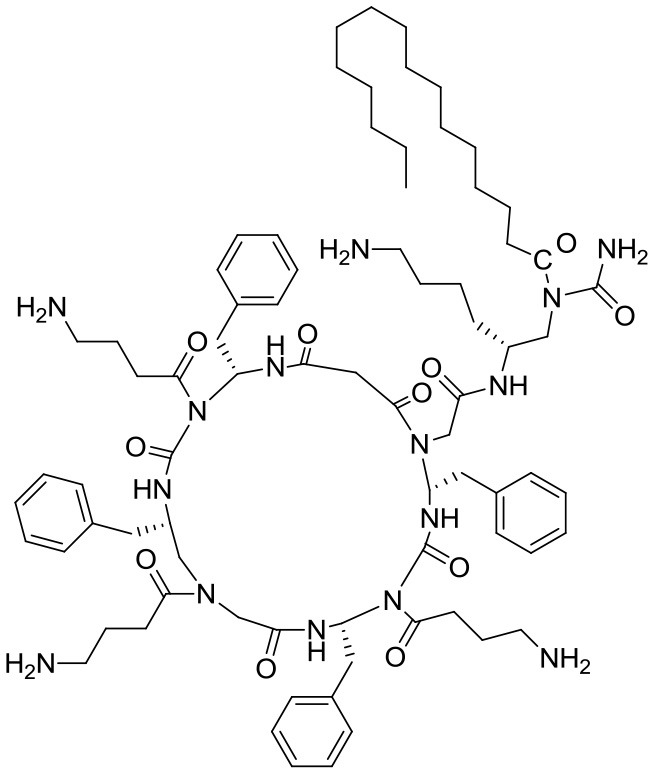 Lipid tails added outside the cyclic rings through an amphipathic *⋎*-AApeptide building block	Not shown	*Pseudomonas aeruginosa* and Methicillin resistant *Staphylococcus epidermidis*	< 5–50/40–80 (YL-36: 6.25–12.56/70–80)	< 5–50/10–70 (YL-36: < 5)	Surfactant-like (micelles)	Membrane disruption	1- >25 (YL-36: 1–5)	(Hemolysis) 100–250 (YL-36: 100)	Padhee et al., 2015
	SAR: Cyclization reduces structure motility and facilitates bacterial membrane disruption, while lipidation encourages their interactions with membranes. Lipid tails may retard the growth of biofilms and form cationic micelles upon interaction with the matrix.										

*Columns: compound ID for the purposes of this paper; peptidomimetic, molecule, or chemical class mimicked by the AApeptide, chemical structure, and molecular weight of the main compound in the studied model; concentration and percentage of action range tested for the antifouling and/or eradication model; antibiofilm and antimicrobial mechanisms of action; minimal inhibitory concentration (MIC); and cytotoxicity. A brief structure activity relationship (SAR) is presented below each AApeptide*.

* *Based on antibiofilm evaluation*.

In order to verify the selectivity of the compounds, cationic residues such as lysine, ornithine, and arginine were added, and the hemolysis profiles assessed. Lysine decreases hemolytic and antibacterial activities, ornithine increases them, and arginine has no effect. Often amphipathic agents are cytotoxic, but at a concentration of 25 μg/mL, the compounds did not show noticeable cytotoxicity against either the HK-2 renal epithelial cell line or the K562 human erythroleukemic one. Table 1 details the most promising AApeptide, “Compound 13,” which was tested for antibiofilm activity at concentrations below the minimal inhibitory concentration (MIC). At this level, a 50–75% reduction of biofilm formation was seen via crystal violet staining in both *E*. *coli* (ATCC 25922) and *A*. *baumannii*.

In another study, Padhee et al. (2015) assessed the biofilm antifouling and eradication activities of peptidomimetics based on the structures of daptomycin and polymyxin B. The main compound, YL-36, is a cyclic γ-AApeptide having both charged and neutral radicals (Table 1, ID 2). YL-36 was designed by joining lipid tails from amphiphilic building blocks with the cyclic rings, with ornithine as cationic residues. Lipo-cyclic structures turn out to have a broader-spectrum of antimicrobial activity than the others, and they work against inflammation by suppressing pro-inflammatory cytokines. With YL-36, 70–80% biofilm antifouling activity was observed with both *P*. *aeruginosa* and Methicillin-resistant *Staphylococcus epidermidis* (MRSE), although these results are inconclusive since the concentrations tested were over the MIC. In any case, the potential for antibiofilm activity could be due to the presence of lipid tails which can retard biofilm formation. The structures that are globally amphipathic probably line up to form micelles when there is an interaction with the polyanionic exopolysaccharide matrix, ultimately disrupting it. Note that YL-36 is not hemolytic, meaning that this compound is highly selective.

### Peptoids as a peptidomimetic strategy

**Figure 2:**
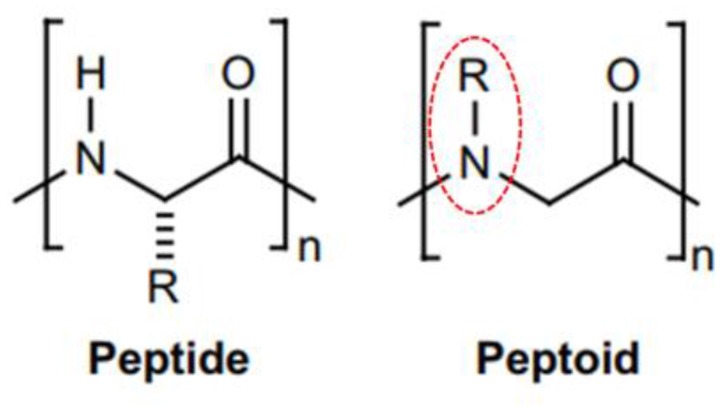
Peptide and peptoid monomer structures differ. (**Left**) Illustration of a classic glycine peptide unit, which has a chiral carbon linked to amino, carboxyl, and radical groups. **(Right)** An *N*-substituted R = H for glycine amino acids residues. This has a radical group linked to the amino group instead of the chiral carbon, identified by dotted circles in red.

Peptoids are oligomers of *N*-substituted glycine units (Figure 2). Their side chains extend from the main-chain nitrogen rather than from the α-carbon, thus yielding secondary structures including helices, loops, and turns. They are achiral foldamer molecules, and retain the functionalities and backbone polarity of peptides (Yoo and Kirshenbaum, 2008; Zuckermann and Kodadek, 2009; Mándity and Fülöp, 2015).

#### Antibiofilm peptoids

Hoque et al. (2015) explored a series of small acyclic amphiphilic peptoids based on AMP structures. They did this by inserting two non-amino acid positive charges, two lipophilic alkyl moieties, and two non-peptidic amide groups (Table 1, ID 3). They demonstrated that antimicrobial activity and hemolytic action correlate to the lipophilic alkyl chain/spacer and increases in chain length, which changes selectivity. The most promising molecule, “Compound 2d” (Table 2, ID 3), shows optimum amphiphilicity, and is able to disperse both *S*. *aureus* and *E*. *coli* mature biofilms at the solid-liquid and liquid-air interfaces, with complete eradication at 32 μg/mL even while biofilm is already formed on the cover slips. In addition, 2d also decreases bacterial viability inside the biofilm, whereas the cell viability of non-treated biofilm increases. Although the study made no mention of an antibiofilm structure-activity relationship (SAR), 2d was non-toxic to human erythrocytes and human kidney cells.

**Table 2:** Summary of chemical and biological information for peptoids.

	**Peptoids**				Concentration^*^(μ**g/mL) / action range (%)**		Mechanism^*^				**References**

**ID**	**Peptidomimetic**	**Chemical structure**	**Molecular weight (M)**	Model^*^	**Antifouling**	**Eradication**	**Biofilm**	**Antimicrobial**	**MIC (**μ**g/mL)**	**Cytotoxicity (**μ**g/mL)**	
3	Acyclic model and the main compound **2d**	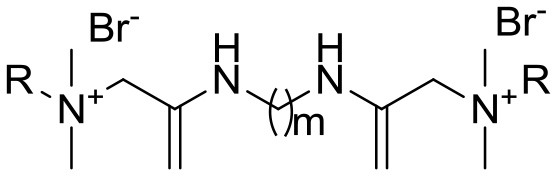 (Compound **2d***: m* = 6 and *R* = C_8_H_17_) Two positive charges, two lipophilic moieties, and two non-peptidic amide groups	Not shown	*Staphylococcus aureus* and *Escherichia coli*		4–64 ~100 (*S. aureus*)	Not shown	Membrane disruption	1.9 (*E. coli*) and 3.9 (*S. aureus*)	(Hemolysis) 780; (HEK293) 220	Hoque et al., 2015
	SAR: Varying the nature of the lipophilic alkyl chain and spacer chain length emphasizes the role of optimum amphiphilicity in the development of non-toxic yet potent membrane-active antibacterials.										
4	Submonomers structures of 1, 1-11_mer_, 1-Pro_9_, 1-achiral, **1-C13**_4mer_, 1_4mer_, 1-*N*ssb	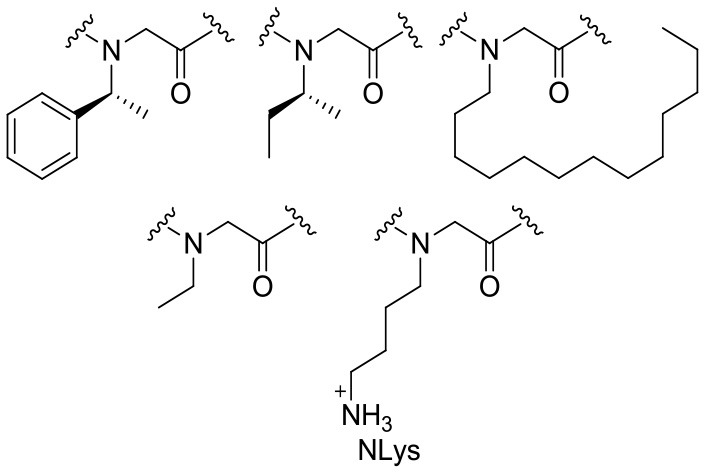 Peptoid submonomers: Alkylated and unalkylated analogs of an amphipathic and cationic dodecamer peptoid	Not shown	*Pseudomonas aeruginosa* (PA14)	< 5–100 μM/40–70	< 5–100 μM/40–60	eDNA, cell-cell detachment/surfactant-like (micelles)	Not shown	12.5->100 μM	Not shown	Kapoor et al., 2011
	SAR: Peptoids can bind extracellular (eDNA) and may facilitate detachment or disruption of otherwise-stable biofilm structures. Oligomerization via interactions with aromatic side chains would increase the concentration of peptoids near the cell membrane, increasing peptoid activity and perhaps also contributing to biofilm detachment. The hydrophobic tail confers a surfactant-like nature that may aide in micelle formation, which could interact with and disrupt the hydrophobic matrix.										
5	Lysine–norspermidine conjugates model	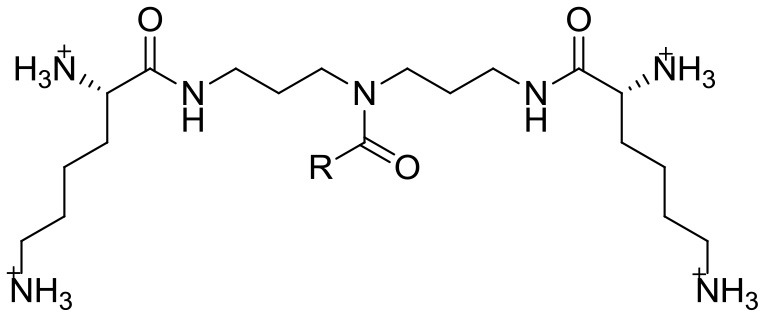 Configuration L,L and *R* = C_9_H_29_ (**1**), C_11_H_23_ (**2**), C_13_H_27_ (**3**), C_15_H_31_ (**4**), C_17_H_35_ (**5**), C_17_H_33_ (**6**), C_17_H_31_ (**7**) Configuration D,D and *R* = C_13_H_27_ (**8**), C_15_H_31_ (**9**) Structures of lipophilic lysine–norspermidine conjugates with trifluoroacetate counterions	Not shown	*Staphylococcus aureus* (MTCC 737)		116–1000 μM/> 80	Electrostatic and hydrogen bonding interactions with the matrix components of the biofilm	Membrane disruption	6	(Hemolysis) 730	Konai and Haldar, 2015
	SAR: D-amino acids such as D-Tyr, D-Leu, D-Trp, and D-Met were also shown to be natural triggers for biofilm disassembly, although none of these possessed significant antibacterial activity. The introduction of four positive charges and hydrogen bond-forming units into a norspermidine backbone would yield greater electrostatic and hydrogen-bonding interactions with the matrix components. In addition, the lipophilic moiety should enhance interaction with the bacterial membrane.										
6	β-peptoid–peptide hybrid oligomers (i.e., 1a−3d) and the mixed amino/guanidino subtype (i.e., 4a−4d)	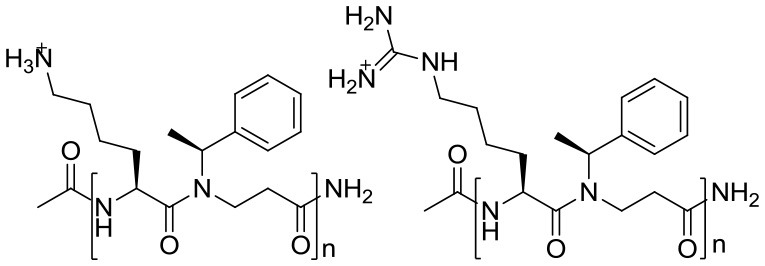 **1 2** 1a (*n* = 5), 1b (*n* = 6), 1c (*n* = 7), 1d (*n* = 8) 2a (*n* = 5), 2b (*n* = 6), 2c (*n* = 7), 2d (*n* = 8) 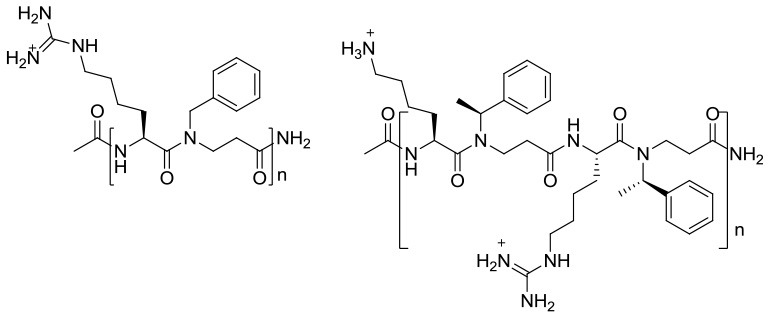 **3 4** 3a (*n* = 5), 3b (*n* = 6), 3c (*n* = 7), 3d (*n* = 8) 4a (*n* = 1), 4b (*n* = 2), 4c (*n* = 3), 4d (*n* = 4) Incorporation of chiral hydrophobic β-peptoids and guanidinylated amino acid side chains while keeping the length relatively short	935–3734 (2b: 2815.84)	Methicillin resistant *Staphylococcus epidermidis* RP62A (ATCC 35984)	1–16 (2b: 4)/40–100 (2b: 100)	8–16 (2b: 8)/~85 (2b: ~85)	Multi mechanisms	Bactericidal	1–4	(Hemolysis) > 500; (HeLa) 46- > 1000 (2b: > 500; 90)	Liu et al., 2013
	SAR: Longer chain length was correlated with increased antimicrobial activity. This tendency was more pronounced in the lysine-containing subclasses (1 and 4) than in the homoarginine-rich ones (2 and 3). A design based on alternating oligomers with only amino or guanidino/amino functional groups in a 1:1 ratio may be a promising strategy to keep cytotoxicity at an acceptable level. Still, some guanidino side chain content is required for antibiofilm activity and chirality appears to be essential for efficient killing planktonic cells.										

*Columns: compound ID for the purposes of this paper; peptidomimetic, molecule or chemical class mimicked by the peptoids, chemical structure and molecular weight of the main compound in the studied model; concentration and percentage of action range tested for the antifouling and/or eradication model; antibiofilm and antimicrobial mechanisms of action; minimal inhibitory concentration (MIC); and cytotoxicity. A brief structure activity relationship (SAR) is presented below each peptoid*.

* *Based on antibiofilm evaluation*.

Kapoor et al. (2011) selected several peptoid analogs to an acyclic amphipathic and cationic dodecamer peptoid (Table 2, ID 4) based on AMP structures. The alkylated peptoids were active against planktonic cells, while the unalkylated ones were not. The 1-C13_4mer_ peptoid (H-*N*tridec-*N*Lys-*N*spe-*N*spe-*N*Lys-NH_2_) is the main compound, preventing about 70% of biomass formation in *P*. *aeruginosa*. In addition, peptoids 1 and 1-C13_4mer_ impaired preformed biofilms by 60 and 40%, and reduced cell viability by about 1.5 and 3 logs, respectively. Antibiofilm activity against *P*. *aeruginosa* was measured at 12.5 μg/mL (MIC) and the biomass was assessed with crystal violet staining. Still, the authors discuss the possibility of using peptoids to bind DNA and facilitate biofilm detachment and/or disruption. Indeed, their activity could be due to their inherent oligomerization via aromatic side-chain interactions. The hydrophobic tails might bestow their efficiency in reducing cell viability, as these confer a surfactant-like nature which causes micelles to form. Micelles may strongly interact with and disrupt the hydrophobic exopolysaccharide matrix, facilitating deeper peptoid penetration into the matrix.

Konai and Haldar (2015) went in the opposite direction. They started with spermidine and norspermidine polyamine structures, both known to have antibiofilm properties. Looking for significant antibacterial activity at minimum biofilm inhibitory concentrations (MBICs), they synthesized a series of acyclic amphipathic conjugate molecules (Table 2, ID 5). These contained a cationic moiety made of various fatty acids acting as lipophilic tails and the amino acid lysine (L-lysine and D-lysine), a known trigger for biofilm disassembly. Crystal violet staining, confocal imaging, and killing curve determination showed that “Compound 8” reduces *S*. *aureus* viability in preformed biofilm in a concentration-dependent manner. However, the best MBIC value (116 μM) was found in derivatives “4” (L,L configuration, *R* = C_15_H_31_) and “9” (D,D configuration, *R* = C_15_H_31_). The mechanism of biofilm disruption is still being investigated, but improved electrostatic and hydrogen bonding interactions with the biofilm's extracellular matrix components may play a major role. The compounds were non-toxic to erythrocytes.

Yang Liu et al. (2013) previously described a synthetic approach to designing acyclic oligomers based on AMP structures with alternating repeats of α-amino acids and β-peptoid residues. The representative compounds for two subclasses are shown in Table 2 (ID 6). These have chain lengths of 4–16 residues, and longer chain lengths often correlated with increased antimicrobial activity within the subclass. This tendency was more pronounced in the lysine-containing groups (1 and 4) than in the homoarginine-rich ones (2 and 3). The hybrid oligomers also inhibited *S*. *epidermidis* biofilm formation and displayed antibiofilm activity against preformed *S*. *epidermidis* biofilm. In comparison with their lysine-containing counterparts (such as 1d and 4c), the fully guanidinylated (e.g., hArg-rich) oligomers (e.g., “2b”) killed slow-growing cells faster, and had more antibiofilm capacity. Chirality appears to be essential for the efficient killing of both slow-growing planktonic cells and biofilms in all the studied oligomers. They were not toxic to erythrocytes, but are toxic to HeLa cells in a concentration-dependent manner. To keep cytotoxicity at acceptable levels, a promising strategy may be to design alternating oligomers that display only a 1:1 ratio of amino or guanidino/amino functional groups.

Finally, since the antibiotic bioactivity that has been explored is usually due to membrane disruption, cationic molecules and bacterial membrane structure-activity relationships have been thoroughly investigated, and amphiphilic molecules probably act in the same way since they act as partial cationics. Moreover, the evaluation of cytotoxicity levels shows that both peptidomimetics and their expected amphiphilicities have good potentials. Finally, both AApeptides and peptoids have been shown to act as effective antibiofilm agents, although their bioactivity and selectivity depend on optimal amphiphilicity. Therefore, we highlight that acyclic conformation and lipid tails, neutral aromatic compounds, and ornithine substituents should be the most advantageous peptidomimetic structural improvements in order to obtain antibiofilm molecules.

## Conclusion

In microorganisms, biofilm lifestyle is a significant virulence factor that results in enhanced resistance to medical treatment (Otto, 2014). This means that antibiotics are less effective, and clearly biofilms have a considerable clinical impact (Del Pozo, 2018). Methods for combatting biofilms using natural peptides seem promising, but their therapeutic relevance is limited by inherently low stability and availability (Kang et al., 2014). Therefore, antibiofilm peptidomimetics are being studied as way to mimic natural peptides while avoiding their drawbacks (Mizuno et al., 2017).

In short, the active structures we discuss tend to mimic naturally occurring antimicrobial molecules such as host defense peptides (HDPs) and antimicrobial peptides (AMPs). These are both endogenous polypeptides produced by multicellular organisms, and they act as an evolutionarily conserved mechanism of innate immune defense (Huang et al., 2014). Thousands of these peptides have been identified in bacteria, plants, insects, birds, fish, and mammals. Family members are highly diverse in their sequences, but generally of small size, made up of 12–50 amino acids. They have similar overall cationic charges of +2 to +9, and are amphipathic, with over 50% hydrophobic residues (Kindrachuk and Napper, 2010). Moreover, it is known that AMPs show broad-spectrum antimicrobial activities and have a low propensity for developing resistance (Seo et al., 2012; Lázár et al., 2018). Peptidomimetics are the same, even though they are all designed to be amphiphilic instead of cationic like AMPs. To confer the desired amphiphilicity, the molecules are linked to different substituents, which are charged and non-charged radicals. The most relevant of those are lipid tails, neutral aromatic compounds, and charged ornithine amino acids.

Although there is an incomplete understanding of the mechanisms of action of antibiofilm peptidomimetics, it seems that their amphiphilicity improves their hydrogen and electrostatic interactions with matrix components, such as those with surfactants.

In the past 40 years, more than 30,000 articles have been published about microbial biofilms (source: PubMed database). This mass of research has been dedicated to understanding biofilms dynamics and to decreasing its effects, but so far no effective treatment has been developed (Bjarnsholt et al., 2013). Antibiofilm AApeptides and peptoids are two very promising families of peptidomimetics for the development and refining of new antibiofilm agents to be used in the fight against resistant microorganisms.

## Author contributions

RG wrote the manuscript with support from SG, AM, and RGB. All authors provided critical feedback and helped shape the manuscript.

### Conflict of interest statement

The authors declare that the research was conducted in the absence of any commercial or financial relationships that could be construed as a potential conflict of interest.
